# Post-Ischaemic Immunological Response in the Brain: Targeting Microglia in Ischaemic Stroke Therapy

**DOI:** 10.3390/brainsci10030159

**Published:** 2020-03-11

**Authors:** Charlotte Rawlinson, Stuart Jenkins, Laura Thei, Mark L. Dallas, Ruoli Chen

**Affiliations:** 1 School of Pharmacy and Bioengineering, Keele University, Staffordshire ST5 5BG, UK; rawlinson.c.t.b@gmail.com; 2 School of Medicine, Keele University, Staffordshire ST5 5BG, UK; s.i.jenkins@keele.ac.uk; 3 School of Pharmacy, University of Reading, Reading RG6 6UB, UK; l.j.thei@reading.ac.uk (L.T.); m.dallas@reading.ac.uk (M.L.D.)

**Keywords:** ischaemic stroke, neuroinflammation, microglia, pro-inflammatory, anti-inflammatory, phenotype

## Abstract

Microglia, the major endogenous immune cells of the central nervous system, mediate critical degenerative and regenerative responses in ischaemic stroke. Microglia become “activated”, proliferating, and undergoing changes in morphology, gene and protein expression over days and weeks post-ischaemia, with deleterious and beneficial effects. Pro-inflammatory microglia (commonly referred to as M1) exacerbate secondary neuronal injury through the release of reactive oxygen species, cytokines and proteases. In contrast, microglia may facilitate neuronal recovery via tissue and vascular remodelling, through the secretion of anti-inflammatory cytokines and growth factors (a profile often termed M2). This M1/M2 nomenclature does not fully account for the microglial heterogeneity in the ischaemic brain, with some simultaneous expression of both M1 and M2 markers at the single-cell level. Understanding and regulating microglial activation status, reducing detrimental and promoting repair behaviours, present the potential for therapeutic intervention, and open a longer window of opportunity than offered by acute neuroprotective strategies. Pharmacological modulation of microglial activation status to promote anti-inflammatory gene expression can increase neurogenesis and improve functional recovery post-stroke, based on promising preclinical data. Cell-based therapies, using preconditioned microglia, are of interest as a method of therapeutic modulation of the post-ischaemic inflammatory response. Currently, there are no clinically-approved pharmacological options targeting post-ischaemic inflammation. A major developmental challenge for clinical translation will be the selective suppression of the deleterious effects of microglial activity after stroke whilst retaining (or enhancing) the neurovascular repair and remodelling responses of microglia.

## 1. Introduction

Ischaemic stroke constitutes about 85% of all stroke events [1]. The underlying pathophysiology of ischaemic stroke is complex and has not yet been fully elucidated. However, mechanisms underlying excitotoxicity, inflammatory pathways, oxidative damage, ionic imbalances, apoptosis, angiogenesis and neuroprotection are widely acknowledged as having a role in ischaemic stroke pathology [2]. The ischaemic cascade is governed by a multitude of molecular events, which variably contribute to the secondary progression of injury in the post-ischaemic phase [2]. With variable lesion-induced plasticity, the degree of impairment post-ischaemia differs dramatically between cases, hindering accurate prognoses [3,4]. As the brain links all of the peripheral organs, brain ischaemia can affect function throughout the body, whereas alterations in peripheral organ function also affect the brain. For example, the immune system is suppressed after ischaemic stroke [5,6,7,8], whereas systemic infection aggravates brain oedema in hypoxia [9]. Up to 95% of stroke patients develop systemic (medical) complications [10,11] and/or neurological complications, as well as stroke syndromes [12,13]. These complications are not only a leading cause of death in stroke patients, with a mortality rate between 23% and 50%, but also pose a major challenge for post-stroke treatment and recovery and may delay or prevent aggressive rehabilitation [10,11,12,13,14,15,16]. 

In the ischaemic brain, microglia, the principal immune cells of the central nervous system (CNS), produce a plethora of pro- and anti-inflammatory mediators, which have critical roles in both exacerbating tissue damage [17] and protecting the brain against ischaemic and excitotoxic injury [18]. In contrast to the rapid resident microglial response, blood-derived leukocytes are usually recruited to the brain parenchyma following a delay of hours to a few days [19]. Therefore, there is an acute therapeutic window in which microglia represent the key targets for immunomodulatory therapies, rather than peripheral infiltrating immune cells. The identification of microglia as a key regulatory factor in ischaemic stroke presents a range of new potential therapeutic targets for neuroprotection [20]. However, the exact contributions of microglia in the context of ischaemic stroke are not yet fully understood. For example, it is unclear whether microglia can generate beneficial effects in both the acute and subacute post-ischaemic phases since neuroinflammation has roles in both promoting recovery and exacerbating secondary neuronal injury [21]. The complex and multiphasic roles displayed by microglia in ischaemic stroke pathophysiology constitute a major challenge to the development of immunomodulatory therapies. The aims of this review article were to describe the pathophysiological roles of microglia following an ischaemic stroke, to summarise the spatio-temporal dynamics of the signalling cascades underlying microglial biology and to describe potential new therapies for ischaemic stroke, based on modulating microglial activation and/or microglial transplantation.

## 2. Microglial Morphology, “Activation” States and Functions in the Brain 

Microglia, comprising ~10% of cells in the brain, are the resident mononuclear phagocytes of the CNS with critical roles in initiating innate and adaptive immune responses [22,23]. Microglia also contribute to the maintenance of homeostasis within the CNS, including promoting neuronal survival and mediating synaptic plasticity [24,25]. These roles involve the secretion of trophic factors, e.g., brain-derived neurotrophic factor (BDNF) [26,27], insulin-like growth factor-1 (IGF-1) [28] and pruning of neuronal synapses, which involves complement factor C1q [29,30,31]. Microglia also remodel the extracellular matrix through the secretion of several proteolytic enzymes, such as matrix metalloproteases (MMPs) and tissue-type plasminogen activator (tPA) [28]. 

Microglia are not homogeneously distributed, with the hippocampus, olfactory telencephalon, basal ganglia and substantia nigra reportedly the most densely populated regions [32]. Regional microglial heterogeneity may have a role in enabling localised homeostatic functions and region-specific sensitivities to microglial dysregulation and involvement in age-related neurodegenerative processes that have neuroinflammatory mechanisms [33,34]. Microglial diversity may be relevant to ischaemic stroke, for which age is a risk factor and neuroinflammation is present [35,36]. Genome-wide transcriptional profiling of microglia from discrete brain regions has found that cerebellar and hippocampal microglia adopt a more immune-vigilant state compared to cortical and striatal regions, and this has been accompanied by relatively greater expression of an extensive set of co-regulated genes involved in energy metabolism. Increased expression of genes involved in the immune response (e.g., *Irf7*, *Stat2*, *Oasl1*, *Sp100*, *Csprs*, *Isg20*, *Ifit* families, *Bst2*, *Zbp1*) has been found in the cerebellum and hippocampus compared to other brain regions. In addition, the microglial transcriptome has been found to age in a non-uniform manner across brain regions [33]. This is an important consideration for the development of microglia transplantation, as the cerebral region from which exogenous microglia are isolated may have a significant impact on their efficacy.

The immunological functions of microglia are mediated by changes in cellular activation status. This has frequently been described in terms of microglial phenotypes, and with reference to polarisation: microglial populations may be predominantly pro-inflammatory (M1) or predominantly anti-inflammatory (M2), representing two poles of activation, with an unactivated phenotype in between (sometimes referred to as M0). These descriptions presumed relative homogeneity within the microglial populations, such that genes/proteins with increased expression in pro- or anti-inflammatory conditions have been touted as markers for M1 or M2. However, recent advances have enabled single-cell transcriptomic analysis of microglia [37]. These data show substantial heterogeneity within populations, with some cells simultaneously exhibiting M1 and M2 markers, and a lack of consistency for M2 markers, such that expression of any individual M2 marker has not been strongly correlated with expression of other M2 markers [37,38]. M1 remains broadly reliable as a description of a pro-inflammatory activation state, but the label M2 has limited utility for accurately indicating a gene/protein expression profile [37]. It is likely the M1/M2 nomenclature for microglia will be replaced in the near future, but the terms are still in widespread use, and so have been used here in the discussion of the literature, with specific reference to relevant stimuli/markers.

**Figure 1:**
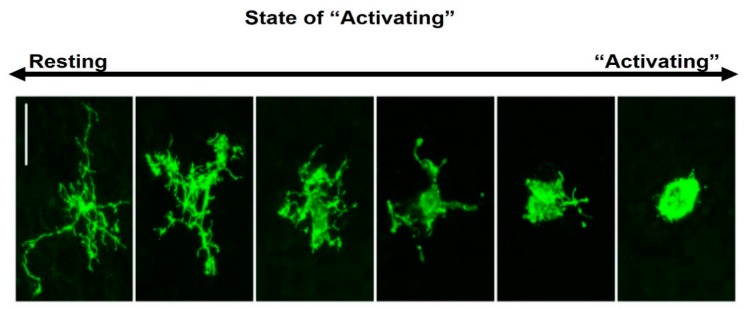
Morphological features of ramified and amoeboid microglia. The resting-state shows a small soma and fine ramified processes (may be referred to as M0, unactivated or homeostatic). Following ischaemia, microglia display changes in morphology, retracting their processes and developing a large (amoeboid) soma. These morphologies are illustrated for Iba1+ microglia in macaque neocortex [43].

Microglial morphology varies with age, sex and tissue region, but is most strongly influenced by disturbances to CNS homeostasis [39]. Under normal homeostatic conditions, microglia exhibit a highly ramified morphology with a small soma (Figure 1). Microglia reside predominantly in grey matter, with these cells expressing more ramifications than those found in white matter [40], where microglia typically orientate along nerve fibre tracts and have elongated soma [41]. Although frequently described as resting, this unactivated phenotype (M0) is highly active, with constantly moving processes surveilling the immediate surroundings [42,43,44]. In response to cues associated with pathogens or tissue damage, microglia typically extend their processes towards these cues [41].

Microglia are highly plastic, with their activation status varying as a disease/injury develops and resolves [45]. Specific stimuli [e.g., ATP, glutamate, cytokines, prostaglandins, zinc, reactive oxygen species (ROS) and heat shock protein (HSP)60] induce microglial proliferation and chemotaxis. ATP is an endogenous agonist for the P2X7 purinergic receptor, activation of which has been suggested as a mechanism for microglial proliferation [46,47,48]. Upon ischaemic injury, feed-forward ATP-induced release of ATP from astrocytes is a possible mechanism for activation of microglial P2X7 receptors [49]. Stimulation of microglial glutamate receptors may also be involved in the induction of microglial proliferation and morphological changes exhibited by microglia in ischaemic conditions [50]. There is evidence that the mammalian target of rapamycin (mTOR) signalling pathway is involved in microglial activation in response to pro-inflammatory cytokines. The mTOR inhibitor RAD001 has reduced microglial proliferation and viability [51]. Peroxisome proliferator-activated receptor–r (PPAR-r) is a subtype of PPARs, which are ligand-activated transcription factors of the nuclear hormone receptor superfamily. Treatment with PPARγ ligands is associated with increased PPARγ expression and reduced microglial activation and migration to the peri-infarct regions [52,53]. Zinc has been shown to induce activation of microglia in culture and in the brain; injection of the zinc chelator CaEDTA prevents ischaemia-induced microglial activation [54]. Reactive species, such as hydrogen peroxide, have also been associated with an enhanced inflammatory response by microglia. Microglial proliferation can be stimulated by several pro-inflammatory mediators that are able to directly stimulate microglial nicotinamide adenine dinucleotide phosphate (NADPH) oxidase, leading to subsequent hydrogen peroxide production, which acts as a mitogenic signal for microglia [55]. Similarly, stimulation of the triggering receptor expressed on myeloid cells 2 (TREM2) by HSP60 activates morphological changes in microglia to induce phagocytic activity [56]. 

The M1 phenotype is characterised by high expression of pro-inflammatory mediators [e.g., interleukin (IL)-1β, IL-6, tumour necrosis factor-alpha (TNF-α)], the immunoglobulin G (IgG) Fc receptors CD16/32 and inducible nitric oxide synthase (iNOS) (Table 1) [57,58]. Pro-inflammatory mediators, released by M1-activated microglia, can initiate neuronal apoptosis and blood-brain barrier (BBB) disruption [58]. M1 microglia also secrete matrix metalloproteinase (MMP), which degrade extracellular matrix proteins and disrupt BBB [40]. Increased BBB permeability promotes cerebral vasogenic oedema, haemorrhagic transformation and the leakage of toxic molecules into the brain, as well as facilitates the infiltration of circulating neutrophils and macrophages [57,58]. Moreover, neurotoxic molecules secreted by M1 microglia can damage neurons and inhibit functional recovery [57].

The putative M2 phenotype is frequently characterised as displaying greater levels of one or more of the following: transforming growth factor-beta (TGF-β), IGF-1, mannose receptor (CD206), arginase 1 (Arg1), chitinase-like 3 (Chil3 or Ym1), IL-10 [40,57]. There is increasing evidence that M2 microglia facilitate tissue repair and remodelling through the production of anti-inflammatory cytokines and neurotrophic mediators, which suppress inflammation and facilitate axonal outgrowth and angiogenesis (Table 1) [59]. For example, Ym1 and Arg1 limit the degradation of extracellular matrix components by MMPs [60]. IL-10 downregulates the production of inflammatory cytokines through negative feedback and upregulates the expression of nerve growth factor (NGF) and glutathione (GSH), which reduce neuronal death via caspase-3 inhibition [61]. Furthermore, TGF-β1 upregulates the expression of anti-apoptotic proteins (e.g., Bcl-2, Bcl-x1), thereby promoting neuronal survival [62]. 

Several studies have described subtypes of M2 activation, based on specific stimuli (singly or in combination) in in vitro conditions. M2a activation is triggered by IL-4 and IL-13, which signal through IL-4Rα to induce a host of downstream processes that lead to potent anti-inflammatory functions, e.g., Arg1 upregulation, inhibition of nuclear factor-kappa B (NF-κB) isoforms and production of scavenger receptors for phagocytosis. Exposure to immune complexes and stimulation of toll-like receptors (TLR) results in M2b activation. M2b microglia have similarities to M1 microglia, e.g., the lack of any M2 specific markers (Arg1, YM1 or FIZZ1), but they do express the typical IL-10^High^, IL-12^Low^ M2 cytokine profile. The higher levels of major histocompatibility complex (MHC) II and CD86 suggest that M2b microglia can stimulate T cells. M2c activation is stimulated by IL-10, glucocorticoids or TGF-β, which activate a phenotype that is involved in tissue remodelling and matrix deposition after inflammation has been downregulated [63]. These proposed phenotypes have not been reliably demonstrated to occur in in vivo. The high degree of overlap between subtypes suggests that microglia exist as a spectrum of phenotypes depending on external stimuli, so we have focused on the M2 phenotype as a whole as a therapeutic target for ischaemic stroke.

## 3. Microglial Responses in Ischaemic Stroke

Following an ischaemic stroke, distinct regions of cerebrovascular pathology can be identified. In the infarct core, brain cells die from necrosis. Neuronal death stimulates the release of damage-associated molecular patterns (DAMPs), including exogenous peptidoglycans, endogenous HSP and non-protein molecules, e.g., ATP and nucleic acid molecules [81]. DAMPs elicit a strong inflammatory response by activating pattern recognition receptors (PRRs), e.g., TLR1-9 [82]. Activation of PRRs induces intracellular signal transduction pathways, thereby modulating microglial activation, e.g., NF-κB signalling pathway, and Toll/IL-1 receptor (TIR)-domain-containing adapter-inducing interferon-β (TRIF) that induces interferon regulatory factor-3 (IRF3) signalling pathway [83]. Microglia express several receptors, which can recognise DAMPs: TLRs, nucleotide-binding oligomerisation domain (nod)-like receptors (NLRs) and the retinoic acid-inducible gene-1 (RIG1)-like receptors (RLRs). Surrounding the ischaemic core, the penumbra is a region of potentially salvageable tissue, where the apoptotic signal pathways are initiated, e.g., mitochondrial release of cytochrome c initiates caspase-mediated neuronal apoptosis [84]. Cells of the penumbra undergo a substantial morphological and metabolic transformation, along with rapid and profound genetic upregulation [2]. Furthermore, glutamate, an excitatory neurotransmitter, is released by neurons in response to ischaemia and activates glutamate receptors on microglia [85]. 

Post-ischaemia, microglia rapidly migrate to the lesion site, proliferate, display altered gene and protein expression profiles and undergo morphological changes [40,43,86]. Microglia develop macrophage-like capabilities, including phagocytosis, cytokine production, antigen presentation and the release of MMPs, which cause disruption of the BBB [40]. Breakdown of BBB allows peripheral macrophages and neutrophils to infiltrate cerebral tissue [40]. The main sources of macrophages infiltrating into ischaemic brain tissue after stroke include microglia-derived macrophages (MiDM) and monocyte-derived macrophages (MoDM). Peripheral monocytes are migrated through the BBB to the ischaemic brain under the action of chemokines and cell adhesion molecules [87].

The post-ischaemic proliferation of microglia peaks at 48 to 72 h after focal cerebral ischaemia, but may last for several weeks [67]. Both CD11b and CD206 have been detected in the mouse brain as early as 6 h after transient middle cerebral artery occlusion (tMCAo) [88], whereas both M1 markers (CD32, CD16, iNOS, CD11b, CD86) and M2 markers (CD206, IL-10, YM1/2, TGF-β, Arg1, CCL22) have been observed in the mouse brain between 1 and 14 d after tMCAo [89]. CD16/32, an M1 marker, has increased over time, whereas CD206, an M2 marker has appeared in the ischaemic core at 24 h, peaking at 5 d, and declining in the striatum by 14 d [89]. Days 2–7 following ischaemia, a phenotypic shift from M2 to M1 microglia has been observed in the infarct core [89]. In the core region, M2 microglia levels diminish after 3–7 d with some remaining in the penumbra. M1 cells have been found to increase in number in the striatum over time and eventually outnumber the M2 cells throughout the second week [86]. This shift in location suggests microglia move from the core once necrotic debris is phagocytosed [88]. In the penumbra, microglia are further activated, proliferate, migrate to repopulate the core. In months following ischaemia, there is evidence of long-term activation of microglia in both the penumbra and the unlesioned tissue, leading to secondary neuronal damage [90]. 

Microglia, which possess a strong phagocytic capacity, can contribute to the removal of necrotic neurons, tissue debris and disabled synapses, thus preventing secondary inflammation and promoting remodelling after stroke [58,81,83]. The elongated shape and high expression levels of F-actin expressed by M2 microglia facilitate phagosome formation and motility [84]. Microglia that exhibit anti-oxidative responses, including suppressing the post-ischaemic level of ROS, and upregulating GSH and heme oxygenase-1 (HO-1), which inhibit oxidation of phosphatidylserine (PS), promote viable neuron repair [70]. In contrast, microglia that possess high levels of ROS have an increased capacity to phagocytose potentially viable neuron [85,87]. 

A key therapeutic aim of future stroke agents would be counteracting the delayed post-acute shift in microglia activity from an M2 phenotype to M1 without causing complete or chronic inhibition of the inflammatory response. Reducing deleterious actions of microglia at the peri-infarct regions may prevent potential damage to healthy BBB endothelium, thereby reducing the potential for peripheral leucocyte infiltration. 

## 4. Pharmacological Modulation of Microglia Activation in Ischaemic Stroke

Currently, there are no pharmacological treatments approved to target ischaemia-induced inflammation in stroke. Microglial modifying agents administered post-stroke could provide a promising therapeutic strategy for ischaemic stroke [57]. A number of pharmaceutical agents aiming to target distinct microglial markers in ischaemia have shown promising results in experimental studies and clinical trials. Considering the high disease burden of ischaemic stroke, drug repurposing of pre-existing pharmaceutical agents may be an avenue for exploration in addition to the development of new therapeutic agents, which specifically target the phenotypic expression of microglia [1]. It is worth noting that many of these agents have been administered orally and could affect both infiltrating peripheral macrophages and endogenous microglia. It has historically been difficult to differentiate between resident microglia and infiltrating macrophages in the parenchyma of the post-ischaemic brain [91]. It is likely the cells, described as microglia in the literature cited below, are in reality a mix of locally recruited microglia and infiltrating peripheral macrophages. These two populations have been treated as one in the following discussion, as we were interested in the neuroinflammatory response as a potential therapeutic opportunity rather than the phenomenological characterisation of microglia per se. 

### 4.1. Minocycline 

Minocycline, a tetracycline-derived antibiotic, is highly lipophilic, so is able to penetrate the BBB [92]. The anti-inflammatory effects of minocycline are thought to occur through the inhibition of iNOS and via the mitogen-activated protein kinase (MAPK) p38 in microglia [93]. Alternatively, other studies have noted inhibition of MMP activity (particularly MMP-9) and apoptotic pathways as other important mechanistic biomarkers for minocycline [94]. Minocycline inhibits the enzymatic activity of MMP-2 and MMP-9 and at low dose is selective for MMP-9. MMP-9 inhibition presents a therapeutic potential for ischaemic stroke since its production by M1 microglia has been associated with demyelination and axonal injury [95]. Anti-apoptotic effects of minocycline are attributed to reduced cytochrome c release from mitochondria and inhibition of the apoptotic drivers, caspase-1 and -3, as well as enhancement of Bcl-2 [96]. Additionally, a decrease in M1-like microglial responses, including IL-1β, ROS, NO and glutamate generation, has also been associated with minocycline treatment [96]. Minocycline has also been found to attenuate expression of inflammatory genes (e.g., *IL-6*, *IL-1β*, *MHC2* and *TLR-2*) and inhibit pro-nerve growth factor production by microglia [96]. 

The efficacy of minocycline has been studied in animal models of acute neurologic injury. Minocycline has been shown to reduce the number of CD68^+^ cells within the peri-infarct tissue at 3 days following photothrombotic stroke and improve neurological outcomes and reduce infarct volume [97]. However, Tsuji et al. reported that minocycline exacerbated hypoxic-ischaemic brain injury [98]. Hanlon et al. found minocycline treatment had no effect on traumatic tissue atrophy or spatial learning deficits in a rat model of abusive head trauma [99]. Furthermore, minocycline-treated rats have demonstrated exacerbated injury-induced memory deficit [99]. These deleterious effects could be in part explained by minocycline’s effect on mitochondrial function. Minocycline impairs mitochondrial function by depleting endogenous mitochondrial magnesium, inducing permeability of the inner mitochondrial membrane, triggering mitochondrial swelling and cytochrome c release and abolishing the calcium retention capacity of rat liver mitochondria [100]. Diguet et al. advocated caution when considering clinical use of minocycline for CNS disorders [101].

To date, a number of clinical trials of minocycline for use in ischaemic stroke have been conducted with varying degrees of success. Minocycline has shown improved functional recovery and reduced disability and dependence at 90 d post-stroke in two randomised single-blinded studies: ‘minocycline in acute stroke’ [102] and ‘efficacy of minocycline in acute ischaemic stroke: a single-blinded, placebo-controlled trial’ [103]. In 2010, the dose-escalation study ‘minocycline to improve neurological functional outcome’ (MINOS) [104] found that minocycline was efficacious at lower doses (3 mg/kg). A total of 72.8% of patients had an modified Rankin Scale (mRS) score of 0–1 equating to very limited symptoms and being able to carry out all usual daily living activities compared to 75% at 4.5 mg/kg, 50% at 6 mg/kg and 41.5% at 10 mg/kg. However, the outcome was not corroborated by the 2013 study ‘intravenous minocycline in acute stroke: a randomised, controlled pilot study and meta-analysis’ [105], which did not find a significant difference in surviving patients free of disability (Mrs ≤ 2) in the patient group receiving intravenous minocycline compared to the control group who received routine treatment. The effect of minocycline on plasma MMP-9 in the MINOS trial and a comparison group of no minocycline was investigated in ‘MMP-9 in an exploratory trial of intravenous minocycline for acute ischaemic stroke’ [106]. In MINOS, intravenous minocycline reduced MMP-9 levels at 72 h compared to baseline routine treatment (tPA, *p* = 0.0022; non-tPA, *p* = 0.0066) and was lower than in the non-MINOS comparison group at 24 h (tPA, *p* < 0.0001; non-tPA, *p* = 0.0019). Plasma levels of MMP-9 were amplified by tPA. High levels of MMP-9 were associated with increased risk of tPA-related haemorrhage and increased neurologic severity. Lower plasma MMP-9 was seen among tPA-treated subjects in the MINOS trial, and, therefore, concomitant minocycline and tPA treatment might be a therapeutic strategy to prevent the adverse effects of thrombolysis via suppression of MMP-9 activity.

A multi-centre randomised, double-blind, placebo-controlled trial, ‘neuroprotection with minocycline therapy for acute stroke recovery trial’ (NeuMAST) [94,107], did not find evidence for minocycline’s efficacy in improving long-term recovery, and the trial was abandoned in May 2013 after an interim analysis. ‘An open-label evaluator-blinded clinical study of minocycline neuroprotection in ischaemic stroke: gender-dependent effect’ [108] studied the neuroprotective properties of minocycline in ischaemic stroke. Oral minocycline administration improved functional outcomes in terms of NIHSS (National Institutes of Health Stroke Scale/Score; higher scores indicate greater impairment) in a 90 d follow up, but efficacy was only demonstrated in male patients. However, the small sample size of 53 patients and the single-blinded nature reduced the reliability of the trial results. Furthermore, the gender-specific outcome of the trial was not supported by the aforementioned study ‘MMP-9 in an exploratory trial of intravenous minocycline for acute ischaemic stroke’ [106], which used females and males at a ratio of 13:10 in the treated group and a ratio of 18:9 in control and, still, demonstrated minocycline’s efficacy in reducing NIHSS. 

### 4.2. Metformin 

Metformin acts by decreasing gluconeogenesis and increasing peripheral utilisation of glucose, thereby improving glucose levels in type 2 diabetics. Metformin has been found to exert neuroprotective effects when given to rodents prior to middle cerebral artery occlusion (MCAO) for a prolonged period (7 d or 6 weeks), but this effect has not been observed when metformin is administered for a shorter duration (1 d or 3 d) [109,110]. Jia et al. [111] concluded that metformin might even have a beneficial effect when given post-stroke as it stimulated adenosine monophosphate (AMP)-activated protein kinase (AMPK) and alleviated stroke-enhanced serum glucose levels. Jin et al. [112] reported increased angiogenesis and neurogenesis and improved functional recovery following metformin treatment post tMCAo. Since AMPK coordinates control of cell growth and autophagy [113], the neuroprotective effects could be due to autophagy induced by metformin [114]. Furthermore, metformin has also been shown to inhibit NF-κB cascade and suppress neuroinflammation [115,116]. Post-stroke, chronic metformin treatment has suppressed the expression of ‘M1’-associated genes (CD32, IL1b, CD16) and enhanced the expression of ‘M2’-associated genes (CD206, Arg1) [58]. A clinical trial in patients with ischaemic stroke showed a significant decrease in NIHSS score in patients who were given metformin [117].

### 4.3. Statins

Statins, which inhibit 3-hydroxy-3-methylglutaryl coenzyme-A reductase, have been shown to inhibit inflammatory cell recruitment, adhesion, and migration [118]. Statins reduce inflammatory biomarkers and inhibit the activation of inflammatory transcription, leading to neuroprotection. Simvastatin has been linked to altered cytokine secretion (IL-1β and TNF-α) and upregulated endothelial NOS (eNOS) [119]. In addition, statins are thought to exhibit antioxidant effects via ROS production inhibition and have beneficial effects on endothelial function, coronary and cerebral blood flow and haemostasis [120,121]. Atorvastatin has been found to promote angiogenesis and enhance functional recovery after stroke by promoting cerebral blood flow [122]. Pre-treatment with rosuvastatin has similar outcomes to minocycline, in terms of improved neurological score and reduced infarct volume [119]. A prospective, non-randomised patient study found that rosuvastatin treatment improved NIHSS scores (OR of 0.04 for NIHSS score of 15 (95% CI, 0.003 to 0.93)) and reduced mortality (OR of 0.20 (95% CI, 0.02 to 1.67)) in intracerebral haemorrhage (ICH) [123]. The mechanism underlying rosuvastatin’s efficacy in stroke may be related to its ability to modulate microglial activation status, upregulate anti-inflammatory cytokines (IL-10) and suppress pro-inflammatory gene expression (IL-1β, TNF-α) [124]. The safety and efficacy of rosuvastatin were investigated in the ‘effects of very early use of rosuvastatin in preventing recurrence of ischemic stroke [EUREKA]’ trial. Diffusion-weighted imaging showed no significant difference in the development of new ischaemic lesions between the rosuvastatin-treated group and the placebo control group. Furthermore, parenchymal/subarachnoid haemorrhage on gradient-recalled echo magnetic resonance imaging occurred less frequently in the rosuvastatin group [125]. A recent multicentre clinical trial—‘stroke treatment with acute reperfusion and simvastatin’ (STARS07), found that statin-tPA therapy was safe but not efficacious in acute stroke [126]. Lovastatin has been found to be clinically safe at doses above the recommended U.S. Food and Drug Administration (FDA) approved dose in phase 1 dose-escalation study. The maximum tolerated dose has been estimated to be 8 mg/kg/day for 3 days after an acute ischaemic stroke [127]. Furthermore, an ongoing phase 2, randomised safety study involving ischaemic stroke patients aimed to compare the effects of placebo or standard dose lovastatin versus short-term high-dose lovastatin [128]. The primary outcome of this study was the occurrence of myotoxicity and neurological outcomes, and effects on inflammatory markers and lipid levels would also be assessed. However, the results of this trial are not yet in the public domain. 

### 4.4. Indomethacin

Indomethacin is a non-steroidal inflammatory drug that is indicated for osteoarthritis and rheumatoid arthritis. Lopes et al. [129] found that indomethacin treatment in rats with endothelin-1 (ET-1)-induced focal striatal ischaemia inhibited microglial activation at 8- and 14-d post-injury. Indomethacin administration was correlated with increased numbers of neuroblasts in the subventricular zone (SVZ) post-stroke mainly at 14 d post-stroke but did not significantly affect neuronal density or increase the neuroblast population in the infarct area. Similarly, Sandu et al. [130] found that indomethacin administration demonstrated neuroprotective efficacy by increasing the survival of penumbral neurons and by decreasing the infarct size after transient focal ischaemia. Indomethacin was also found to decrease the ED1^+^—activated macrophages/microglia in the infarct core in the young rats and increased the number of proliferating microglia in the peri-infarct region. Bok et al. [131] concluded that indomethacin significantly attenuated CD68-positive microglial activation in a mouse model of focal cerebral ischaemia. Comparably, indomethacin has been found to enhance neurogenesis in rats exposed to focal ischaemia: increased numbers of bromodeoxyuridine (BrdU) positive cells of all lineages have been found, with reduced microglial/monocyte activation [132].

### 4.5. Noggin

Noggin has been shown to prevent brain atrophy and improve functional outcomes through the inhibition of bone morphogenetic protein signalling during the later phases of cerebral ischaemia in mice [133]. Noggin has also been found to augment brain repair processes, including resolution of scar formation, axonal sprouting and angiogenesis, as well as to increase microglial activation and modulate microglial phenotypes to induce a switch from M1 to M2 [134].

### 4.6. PPAR-R

Activation of PPAR-r results in insulin sensitisation and enhances glucose metabolism [135]. PPAR-r has the ability to inhibit the expression of inflammatory cytokines and direct the differentiation of immune cells towards anti-inflammatory phenotypes. 1,25-D3 pretreatment activated PPAR-r, reduced NF-κB and TNF αexpression, improved neurological functions in the rat after tMCAo [136]. Activation of PPAR-r has also been shown to inhibit the expression of iNOS and inflammatory cytokines and to reduce ROS formation [133]. A review of five random-control trials, which investigated the effects of PPAR-r agonists in the prevention of stroke recurrence, was conducted. Although PPAR-r agonists have reduced the recurrence of stroke, their effects on adverse events remain uncertain. However, due to the small number of included studies, limited quality of available study data and the fact that the data was not meta-analysed, the results of the review should be interpreted with caution [137]. 

### 4.7. TNF-α Antagonists

Tumour necrosis factor (TNF) is an immune signalling molecule, centrally involved in ischaemic stroke pathology through its modulation of microglial activation, role in synaptic dysfunction and induction of depressive symptoms and neuropathic pain. Etanercept, a recombinant TNF receptor fusion protein and potent TNF inhibitor, has been found to produce an immediate, significant and sustained improvement in neurological outcomes in patients with chronic neurological dysfunction present for more than 3 years following acute brain injury [138]. This is consistent with a study by Wu et al. [139], in which etanercept markedly reduced cerebral infarct, BBB disruption and neurological motor deficits in rats subjected to tMCAo, and this effect was more pronounced when combined with alpha-lipoic acid. A greater decrease in the serum levels of TNF-α, as well as the brain levels of microglial activation, was observed with the combined drug treatment compared to the separate administration of the drugs. Banno et al. [140] found that edaravone significantly suppressed the production of NO and ROS by activated microglia, though it did not suppress the production of inflammatory cytokines. Edaravone significantly suppressed neuronal cell death and dendrotoxicity induced by either the peroxynitrite donor N-morpholinosydnonimine or activated microglia in a dose-dependent manner. Edaravone has been shown to scavenge free radical in acute ischaemic stroke patients in Japan [141]. However, Isahaya et al. [142] found no significant differences in serum concentrations of TNF-α between ischaemic stroke patients treated with edaravone compared to controls. Nevertheless, edaravone was found to significantly suppress circulating MMP-9 in patients with acute ischaemic stroke. MMP-9 is involved in the pathogenesis of vasogenic brain oedema due to its effect on vascular endothelial permeability, thus offering a further mechanism underlying the efficacy of edaravone in stroke. 

### 4.8. Fingolimod

The sphingosine-1-phosphate regulator (S1P), fingolimod, approved by the FDA in 2010 for the treatment of Multiple Sclerosis was studied in phase II clinical trial for the treatment of stroke [143]. Fingolimod is thought to exert its mechanism by binding to the S1P1 receptor and inhibiting downstream signalling pathways. Signal transducer and activator of transcription 3 (STAT3) inhibition has been suggested as a possible mechanism underlying fingolimod’s efficacy. STAT3 ordinarily elevates NF-κB and pro-inflammatory gene expression. Fingolimod may also downregulate histone deacetylase (HDAC) activity, which further suppresses NF-κB activation, resulting in the downregulation of pro-inflammatory cytokines (e.g., TNF-α, IL-1β), and promotes the expression of neurotrophic factors (e.g., BDNF and glial cell-derived neurotrophic factor (GDNF)), which aid neurogenesis. The lipophilic nature of fingolimod enables it to pass through the BBB, and there is evidence that fingolimod can alter the function of brain cells [144].

### 4.9. Colony Stimulating Factor Receptor Inhibitors

Colony-stimulating factor receptor 1 (CSF1R) is activated by CSF1 (macrophage CSF) or IL34 and is expressed by cells, including monocytes, dendritic cells and peripheral macrophages. Within the CNS, CSF1R is reportedly predominantly expressed in microglia, in contrast to other cell types [145]. CSF1R inhibitors ablate microglia and have recently been used to suppress microglia reactivity in different conditions [146]. CSF1R inhibitors, such as PLX3397, BLZ945 and GW2580, have been administered orally to deplete microglia in the CNS [147]. PLX3397 transiently eliminates ~99% of microglia in the adult mouse brain without any detectable behavioural or cognitive impairment, and the microglia population recovers after cessation of CSF1R blockade [148,149]. CSF1R inhibition reduces neuroinflammation, leading to improved disease phenotype in mouse models of Alzheimer’s disease [150,151,152]. However, Szalay et al. [153] demonstrated that selective elimination of microglia in mice led to a striking 60% increase in infarct size following tMCAo, which was reversed by microglial repopulation. CSF1R inhibition has been reported to exacerbate post-ischaemic outcomes in a mouse model of brain ischaemia, through increased production of pro-inflammatory cytokines [154]. Microglia depletion by long-term treatment with a CSF1R inhibitor, PLX5622, has increased the numbers of neutrophils and enlarged the ischaemic lesion [155]. These adverse responses to experimental depletion in microglial activity highlight the beneficial role of microglia in ischaemic stroke.

## 5. Cellular Therapies for Stroke That Target Microglia

Ischaemic injury in the brain is believed to be mediated by several mechanisms, thus supporting research into a multi-target therapeutic strategy, such as using cell-based therapies involving bone marrow-derived mesenchymal stem (BMSCs) or the direct administration of microglia [156]. Translocator protein (TSPO) has been used as a non-selective biomarker of microglial activation in positron emission tomography (PET) to show the proportion of systemically administered microglia, which are able to cross the BBB [143]. The adhesion receptor macrophage-1 antigen (Mac-1) mediates the adhesion of microglia to the endothelial surface, thus providing a possible mechanism for the infiltrating properties of microglia across the BBB [157]. Mac-1 is a β2 integrin that is constitutively expressed on the surface of microglia. Mac-1 upregulation after oxygen-glucose deprivation (OGD) preconditioning has been found to enable microglia to cross the BBB and reach the rat brain parenchyma [69]. In addition, a study in C57BL/6 mice revealed that M2-like macrophages were able to infiltrate the ischaemic hemisphere via the choroid plexus-cerebrospinal fluid (CSF) route, thus suggesting that autologous transplantation of M2-like microglia into the CSF might be a promising treatment strategy for ischaemic stroke [158]. Apart from the epithelial cells of the choroid plexus, there are two other routes for leukocyte migration from the blood into the brain parenchyma, i.e., BBB and the meningeal blood circulation, each of which could be exploited by microglia [159,160]. 

Studies on direct administration of primary microglia and cell-line microglia in animal models have demonstrated improved axonal outgrowth, reduced infarct size and improved functional outcomes post-stroke. Imai et al. [161] concluded that administration of exogenous microglia after ischaemia protected against ischaemic injury in vivo and improved ischaemia-induced learning deficits. Ekdahl et al. [162] found that microglia pre-conditioned to a protective phenotype could support neurogenesis, progenitor proliferation and survival, migration and differentiation of newly formed neurons in the adult brain following stroke. Kanazawa et al. [69] found that intrathecal administration of primary microglia preconditioned in OGD conditions promoted the secretion of remodelling factors into the brain parenchyma, e.g., vascular endothelial growth factor (VEGF), TGF-β and MMP-9, thus facilitating axonal outgrowth and angiogenesis in the ischaemic cortex of rats. Administration of M1-polarised microglia has exacerbated OGD-induced neuronal death, manifested by reduced microtubule-associated protein-2 (MAP2) expression and increased lactate dehydrogenase (LDH) release, compared to microglia expressing differing inflammatory profiles. Similarly, HMO6 human microglial cell transplantation has significantly reduced ischaemic deficits and apoptotic events in rats exposed to MCAO. The results have been mediated by modulation of gliosis and neuroinflammation, as well as neuroprotection provided by neurotrophic factors and anti-inflammatory cytokines of endogenous and transplanted microglia [161]. However, it is still not known whether functional recovery is directly due to the transplanted cells or whether the cells induce peripheral immune cells to alter cytokine release [163].

In clinical trials, however, Chernykh et al. [164] found that intrathecal autologous M2 macrophage transplantation in nonacute stroke patients did not significantly affect cytokine production. However, 75% of patients exhibited an improved NIHSS score by ≥3 versus 18% in the control group. Furthermore, responder patients had lower spontaneous production of IL-10, fibroblast growth factor-β (FGF-β), platelet-derived growth factor (PDGF) and VEGF and higher stimulation indexes of IL-1β, TNF-α, IFN-γ and IL-6 than non-responders, thus suggesting that the improved neurological activity associated with autologous M2 cells might be mediated through the immunomodulatory activity of M2 macrophages. In contrast, a phase II, randomised controlled trial, in which subacute ischaemic stroke patients received BMSCs (a mean of 280.75 million at a median of 18.5 d after stroke onset), concluded that whilst an intravenous infusion of BMSCs is safe, there was no beneficial effect on stroke outcome [164]. Further studies should seek to establish if a dose-effect relationship exists, as the failure could be due to an insufficiency in the dose administered. It is also possible that at the time of administration of BMSCs at a median of 18.5 d after stroke onset, the amount of cerebral damage may have already reached its peak. In addition, this trial used the intravenous route to administer cell therapy. The relative efficacy of this route of delivery should be compared to other routes, such as the intra-arterial or intracerebral route [165]. Yang et al. [166] concluded that intravenous administration of stem cells in rats with tMCAo achieved a similar functional outcome post-stroke to intra-arterial administration. The lack of clinical efficacy in human studies may also be attributable to the non-polar status of the BMSCs. Hu et al. [89] found that when microglia of different phenotypes were mixed with neurons in a 1:10 ratio prior to administration, both M2 and non-polarised microglia promoted survival of cortical neurons following focal cerebral ischaemia, suggesting a cell-cell contact-mediated protective mechanism.

## 6. Challenges for Stroke Therapies Targeting Microglia 

Since much of the research on microglia “activation” is performed in vitro, interpretation and generalisation of the results must be done with caution. Many pre-clinical studies use microglia derived from neonatal tissue and matured in culture-medium, which does not replicate the complex in vivo maturation process [57]. The culture medium is typically supplemented with 10% (bovine) serum in experimental studies, whereas endogenous microglia are not exposed to serum components, given an intact BBB [167]. It is widely accepted that, in the brain, microglial activity is controlled by inhibitory inputs (e.g., CX3CL1, CD200, CD22 and CD172), which are typically absent in culture. In animal models, the genetic removal of even just one of the factors inhibiting microglial activation dramatically alters the activation profile of microglia and, in many instances, has demonstrated inappropriate activation, including toxic responses. As a result, it must be considered that the absence of these factors in vitro could have a significant impact on the activation status of cultured microglia [168]. 

Laboratory experimentation may generate different activation profiles from those found in live human brain tissue following a stroke, as in vitro cultures commonly demonstrate amoeboid morphologies and are generally considered to exhibit signs of low-level inflammatory activation [169]. Therefore, it may be more scientifically sound to caution against attempts to classify microglial populations as exhibiting a distinct phenotype and instead view microglial “activation” as specific to individual cells [60]. The classification of microglia is currently under discussion with Ransohoff, arguing that the elucidation of regional and age-dependent microglial profiles does not support the notion that microglia will adopt a single state transcriptomic profiles (relating to M1 and M2) under physiological or stress conditions [168]. Whilst frequently portrayed as a single event or a homogenous phenotype, activation of microglia post-ischaemia may be more accurately depicted as a continuum of events, with the heterogeneity of marker expression among the microglial population [44]. 

One of the fundamental hurdles in existing research targeting microglia is that the underlying mechanisms regulating microglial activation are not yet fully understood. Whilst an abundance of pathways and mediators have been identified as regulators of macrophage activation, the assumption that microglial activation is regulated by the same mechanisms cannot necessarily be made. The majority of these signalling pathways appear to overlap and seem to work synergistically with one another, as opposed to functioning independently. In addition to this, the aforementioned spatio-temporal dynamics of microglial activation must also be accurately mapped in order to establish the optimum time to administer therapy. 

Although experimental stroke treatments continue to fail in human clinical trials, the inflammatory/immune responses after cerebral ischaemia are shared between the animal models and patients [170]. Combined with complementary knowledge from in vitro studies, animal models can provide valuable insight into stroke pathology at a system level [171]. A deeper understanding of the in vivo physiological changes post-ischaemia is required to identify further treatment targets for stroke. Current research suggests that upon “activation”, microglia potentiate BBB disruption, thus exposing the brain to systemic responses, such as peripheral infiltration of leukocytes into cerebral tissue [40]. Understanding the mechanism by which microglia increase BBB permeability could provide a focus for future research on improving drug delivery into the ischaemic brain [172].

## 7. Conclusions

Although progress continues to be made in the prevention, treatment and rehabilitation of those affected by stroke, there remains a significant capacity for improvement. With such a complex and multifaceted pathophysiology, targeting the mechanisms by which stroke is known to cause cerebral damage remains a significant barrier to achieving pre-stroke functionality. At present, many patients who survive an ischaemic attack experience a decline in their quality of life, developing conditions, such as hemiplegic paralysis and aphasia [1]. Microglial biology plays a pivotal role in the pathophysiology of neuroinflammation induced by ischaemic stroke, thus presenting an attractive treatment target for pharmacological intervention. However, pharmacological treatments targeting microglial activation remains sub-optimal. A greater understanding of the heterogeneity of phenotypes at a cellular level, as well as the functional impacts of these on a regional level, could inform the development of novel therapies and perhaps improve current treatment regimens, thereby alleviating post-ischaemic damage whilst promoting the reparative functions of microglia [173]. Stimulating cerebrovascular tissue repair may be achievable by targeting distinct microglial populations, focusing heavily on the temporal and spatial aspects of the post-ischaemic response. Modulation of microglial activation status or transplantation of exogenous microglia pre-conditioned with specific stimuli may reverse the neuronal loss and repair neural networks, thus presenting a therapeutic approach to prevent functional disability in stroke survivors. Harnessing the regenerative role of microglia, whilst avoiding the deleterious effects of total immunosuppression, merits further investigation.

## Figures and Tables

**Table 1:** Roles of Microglia in Ischaemic Stroke.

**Biological Process**	**Mediators**	**Effects Potentially Beneficial Post-Ischaemia**	**Effects Potentially Deleterious Post-Ischaemia**
Pro-inflammatory	TNF-α	Possibly neuroprotective; exacerbated infarct in TNF-α^-/-^ KO mice [64]	Exacerbated infarct volume, oedema [58,65]
	Inflammasome		In ischaemic conditions, NLRC4 inflammasome complex induces pyroptotic microglial death, exacerbating inflammatory damage [66] NLRC4^-/-^ KO mice have lesser neurological deficits post tMCAo [67] NLRP3^-/-^ KO mice have lesser BBB breakdown, infarct size, oedema, neurological deficits post tMCAo [68]
	IL-1β, IL-6, IL-12, IL-23, IFN-γ		Prolonged/heightened inflammation. Bystander tissue damage [66]
Anti-inflammatory	TGF-β, IL-4, IL-10, IL-13	Reduced inflammatory damage. Pro-regeneration. Upregulated Bcl-2, Bcl-x1. Enhances dendritic spine formation and synaptogenesis in cultured neurons and provides negative feedback in the production of inflammatory cytokines. Inhibit the activity of caspase-3, upregulate the level of GSH and NGF [69]	
Chemotaxis	CD11b, CD16, CD32, CCL2/MCP1	Facilitate microglial migration to injury sites [70]	CCL2^-/-^ KO mice show decreased injury post-ischaemia [59]; anti-CD11b antibody reduced infarct size in rat tMCAo [71]
Phagocytosis	CD11c (ITGAX)	Clears damaged cells, neurotoxic molecules and molecules inhibitory to repair. Early infiltrating macrophages are CD11c^−^, but CD11c^+^ macrophages may outnumber CD11c^+^ microglia by 3 d post-stroke [63]. Dendritic cells are CD11c^+^ [72]	Microglia may phagocytose damaged neurons, which could otherwise have recovered [73,74]
ROS, RNS	iNOS, NO^−^		Disrupts BBB, facilitating infiltration of peripheral immune cells and toxic molecules from serum. Oxidises PS on the surface of neurons and promotes neuron loss through phagocytosis. Exacerbates glutamate excitotoxicity. Damaging to oligodendroglia.
Anti-oxidants	GSH, HO-1	Inhibit oxidation of PS and promote neuron repair by reducing the phagocytic capacity of microglia [59]	
ECM-degrading enzymes	MMP-3, MMP-9		Degrade extracellular matrix proteins, reduce the integrity of BBB; MMP-9 upregulated by signals from serum [75]
Angiogenesis	VEGF, BDNF, progranulin, MMP-9	Facilitate axonal outgrowth and angiogenesis. MMP-9 degrades chondroitin sulphate proteoglycan, a component of the EC matrix that is reported to inhibit axonal growth [59]	Microglia express VEGF receptor; VEGF is associated with increased BBB permeability [76]
Neurotoxic molecules	Glutamate		Neurotoxic; microglia upregulation of GluR2-4 AMPA receptors is associated with axon and oligodendrocyte damage [77]
Neuroprotective molecules	bFGF	Promotes mitosis of oligodendrocyte precursor cells [78]	
	IGF-1, GDNF, thrombospondins, erythropoietin	Promote plasticity [79]; thrombospondins may be more highly expressed in macrophages [80]	
	BDNF	~30% OX42^+^ cells expressed BDNF post-ischaemia; associated with plasticity (neuronal regeneration) [79]	

Arg1, Arginase 1; BDNF, brain-derived neurotrophic factor; bFGF, basic fibroblast growth factor; GDNF, glial cell-derived neurotrophic factor; GSH, glutathione; HO-1, heme oxygenase; IGF-1, insulin-like growth factor 1; IL-1β, interleukin-1β; IL-13, interleukin-13; IL-4, interleukin-4; IL-6, interleukin-6; IL-10, interleukin-10; IL-12, interleukin-12; IL-23, interleukin-23; iNOS, inducible nitric oxide synthase; MMP-3, matrix metalloproteinase-3; MMP-9, matrix metalloproteinase-9; NGF, nerve growth factor; NLRC4, neuronal apoptosis inhibitory protein (NAIP)/NOD-like receptor 4; NO, nitric oxide; PS, phosphatidylserine; ROS, reactive oxygen species; TGF-β, transforming growth factor-β, tMCAo, transient middle cerebral artery occlusion. TNF-α, tumour necrosis factor- α; VEGF, vascular endothelial growth factor.
